# Assessing the economic cost of road traffic crashes in Saudi Arabia: potential savings from scaling up interventions

**DOI:** 10.3389/fpubh.2025.1637609

**Published:** 2025-09-23

**Authors:** Garrison Spencer, Mohammed Alluhidan, Richard Reithinger, Reem F. Alsukait, Radoslaw Czapski, Adwa Alamri, Volkan Cetinkaya, Fatimah Alshehri, Said Dahdah, Mariam M. Hamza, Christopher H. Herbst, Suliman Alghnam

**Affiliations:** ^1^RTI International, Washington, DC, United States; ^2^Department of Health Economics, Saudi Health Council, Riyadh, Saudi Arabia; ^3^World Bank Group, Washington, DC, United States; ^4^Department of Injury Prevention, Saudi Public Health Authority, Riyadh, Saudi Arabia; ^5^Public Health Intelligence, Saudi Public Health Authority, Riyadh, Saudi Arabia

**Keywords:** road traffic crashes, cost analysis, economic cost, road safety interventions, Saudi Arabia

## Abstract

**Objective:**

This study aimed to (1) assess the direct and indirect economic cost associated with road traffic crashes (RTCs) in Saudi Arabia in 2022; and (2) estimate the potential economic savings associated with scaling up the full implementation of three priority road traffic safety interventions (i.e., speed limit enforcement, seat belt enforcement, and a graduated licensing system for new drivers).

**Methods:**

A cost-of-illness approach was used to estimate the economic impacts of RTCs in Saudi Arabia in 2022. To estimate how scaling up the three priority interventions to improve road traffic safety in Saudi Arabia would impact the estimated economic costs of RTCs, we applied the International Road Assessment Program method to the data and modeled the interventions’ full enforcement.

**Results:**

In 2022, the estimated cost of RTCs was Saudi Riyal (SAR) 137.6 billion (USD 36.7 billion), equivalent to 3.3% of Saudi Arabia’s GDP. Indirect costs account for 70.2% of the total cost of RTCs, with morbidity-related costs driving most of the cost at 40.2%. Men make up the majority (88%) of road traffic injuries and fatalities in Saudi Arabia, resulting in a significant proportion of the total costs. We estimate that scaling up the three priority interventions could prevent 610 deaths and 3,823 injuries per year, resulting in total annual savings of SAR 14.6 billion (0.35% of GDP).

**Conclusion:**

The study underscores the significant costs associated with RTCs in Saudi Arabia and emphasizes the potential substantial economic savings achievable through scaling up existing interventions.

## 1 Introduction

In 2021, road traffic crashes (RTCs) killed nearly 1.19 million people; they are the twelfth leading cause of deaths globally, accounting for more deaths than HIV/AIDS, tuberculosis, or malaria (1, 2). At their current rate, RTCs are likely to become the fifth leading cause of mortality worldwide by 2030, and they are already the leading cause of death among youth aged 5–29 years (2). RTCs injure 20–50 million people annually and are the leading cause of trauma admissions worldwide (3). Furthermore, RTCs cause significant socioeconomic losses, with estimates suggesting a global economic loss of US$ 1.3 trillion between 2015 and 2030 due to RTCs, and costing most countries the equivalent of 3% of their GDP (3). As part of a global commitment to improving road safety, in 2020 the UN General Assembly’s launched the Global Plan for the Decade of Action for Road Safety with the goal of reducing road traffic deaths and injuries by half by 2030 (1).

In Saudi Arabia, RTCs have been a leading cause of morbidity and mortality over the past decade (2, 4, 5). Fatalities due to RTCs in Saudi Arabia are estimated to be 2.3 times greater than the average rate of other high-income countries, and 1.2 times greater than the global average (2). In 2016, Saudi Arabia initiated a National Transformation Plan (Vision 2030), which aims to achieve six overarching objectives, including improved health, employment, and a diversified economy (6). One of the priorities of Vision 2030 is to reduce road traffic injuries to less than 10 per 100,000 by 2030 (6). Several initiatives were adopted to adhere to existing traffic legislation and improve the implementation of stricter traffic safety interventions, including the use of cameras to reduce speeding, increase seatbelt use, and reduce mobile phone use while driving (6–9). These actions are spearheaded by the Ministerial Committee of Traffic Safety (MCTS), which coordinates seven ministries that have responsibilities over different aspects of road safety and oversees the implementation of the national road safety strategy (6, 9). MCTS’s leadership of road traffic safety evidence-based interventions has achieved substantial progress between 2016 and 2021 (10). Thus, the number of RTC fatalities declined by nearly half from 28.8 per 100,000 in 2016 to 18.5 per 100,000 in 2021 (11). Additionally, the number of injuries declined by nearly 25% in the last 3 years from 32,910 in 2019 to 24,446 in 2022 (11).

While significant progress has been made since 2016, the death rate from RTCs in Saudi Arabia remains over double the average of other high-income countries (2, 12, 13). This continues to represent a substantial and avoidable drain on the Saudi economy, including through reduced workforce participation from injuries and deaths, healthcare costs, property damage, and other costs. RTCs can strike individuals of any age—however, unlike other leading causes of death, most mortality and morbidity from RTCs in Saudi Arabia occur before the age of 50 (13). Furthermore, RTCs can leave victims with catastrophic healthcare expenditures, cause early retirement and permanent disabilities, and result in trauma for families and communities.

The objective of the study was to estimate the current economic costs of RTCs in Saudi Arabia, and assess how the enforcement of currently implemented road traffic safety interventions saves lives, averts injuries, and yields economic savings.

## 2 Materials and methods

We used a cost-of-illness approach (14) to estimate the economic impacts of RTCs in Saudi Arabia in 2022. Unlike cost benefit or cost effectiveness analyses, the aim of cost-of-illness studies is descriptive: to itemize, value, and sum the costs of a particular problem with the aim of giving an idea of its economic burden that illness imposes on society as a whole. Under the cost-of-illness approach, economic impacts are divided into direct and indirect costs (14). In this study, direct costs consisted of healthcare costs (i.e., treatments for deaths, severe injuries, moderate injuries, and minor injuries), rehabilitation costs (from severe, moderate, and minor injuries), and property damage costs. The indirect costs included in this study include the economic value of RTC fatalities, the economic value of RTC injuries, and absenteeism costs (due to hospitalization from severe, moderate, and minor injuries). The above does not encompass the entirety of costs that accrue as a result of RTCs, but ones that can be estimated with existing data sources. Other potential costs that are not accounted for in our analyses include productivity losses due to physical disabilities of people involved in RTCs (i.e., drivers, passengers, pedestrians), mental health of people involved in RTCs and their family members, traffic jams resulting from RTCs, legal costs resulting from litigation regarding RTCs, and other administrative costs such as the police response and insurance costs. Consequently, our estimates represent a conservative estimate of the total economic costs of RTCs in Saudi Arabia.

### 2.1 Data sources and analysis

Estimates for the burden caused by RTCs in Saudi Arabia are not consistent across national and international sources. Saudi’s Arabia’s Ministry of Health estimates mortality attributable to RTCs to be 5,754 and 4,555 deaths in 2019 and 2022, respectively (13). The number of RTC-related injuries was 32,856 in 2019 and 24,446 in 2022. WHO estimates that Saudi Arabia’s mortality rate due to RTCs for 2021 was 6,735 deaths (i.e., 18.5 deaths per 100000 population), while the Global Burden of Disease (GBD) study provides a figure of 21,316 deaths for 2019 (2, 12, 15).; GBD estimates the number of RTC injuries in 2019 to be over half a million. Because it has been shown that the GBD study overestimates outcomes related to RTCs (16), guidance from the International Road Assessment Programme (iRAP) recommends a ratio of RTC deaths to injuries of 10:1, which would estimate a RTC-related morbidity (57,540) that is nearly double than the one reported by the Saudi Ministry of Health (17).

The lower number of injuries reported by the Saudi Ministry of Health is likely due to only including RTC-related injuries requiring hospitalization of 24 h or more; additionally, some health services are not covered by the Ministry of Health, with injured individuals not seeking services at all or accessing services in the private sector (where they may not necessarily be reported as RTC-related).

To be conservative in our estimates, the Saudi Ministry of Health data was used for our baseline calculation of the number of RTC deaths and injuries. Because the number of injuries is below what would be expected given the number of deaths from RTCs, we performed a sensitivity analysis using the number of injuries based on the 10:1 guidance from iRAP (17), as well as the number of estimated fatalities reported by WHO (2).

Morbidity data was disaggregated by severity, using the proportions of RTC hospitalizations in each category reported by Alghnam et al. in a retrospective chart-review study conducted at King Abdulaziz Medical City (KAMC) (8). The KAMC hospital is one of Saudi Arabia’s most advanced and specialized trauma centers. It has a capacity of over 1501 beds and another 132 beds in the emergency department; the hospital’s capacity and standards make it comparable to a level-I trauma center in the United States. Injuries were considered minor, when the injury severity score (ISS) was between 0 and 15; moderate when the ISS score was between 16 and 25; and severe when the ISS score was over 25 (8). The study found that of all RTC injuries, 73% were minor (ISS 0–15), 16% were moderate (ISS 16–25), and 11% were severe (ISS > 25) (8).

Applying an arithmetic approach, our analysis calculated the costs of injuries and deaths, the effect of different interventions, and subsequent numbers of lives saved in Microsoft Excel® (Microsoft Corporation, Redmont, WA), using custom, inter-linked worksheets that were set-up for different input and output variables, as well as study outcomes. For our analyses, in as much as possible, we used publicly available data from official Saudi sources, such as the Saudi Ministry of Health and the General Authority for Statistics (GASTAT), and from studies conducted in Saudi Arabia and reported in the peer-reviewed literature. A summary of data sources used in our calculations is shown in Appendix A (8, 13, 18–24), and the number of injuries by severity is provided in Appendix B.

### 2.2 Estimation of direct costs at baseline

#### 2.2.1 Medical costs

Direct medical costs included in the analysis represent the sum of hospital treatment costs, rehabilitation costs, and property damage costs. To calculate treatment costs of road traffic injuries and fatalities, hospital treatment costs for minor, moderate, and severe injuries as reported in the literature and the treatment cost for those who die in hospital were added together. For average treatment costs, adjusted for minor (ISS 0–15), moderate (ISS 16–25) and severe (ISS > 25) injuries, we used figures reported by Alghnam et al. (8). Results were reported in 2018 Saudi Riyal (SAR) and adjusted to 2022 values using consumer price index data from the GASTAT. The cost for those who die in the hospital following an RTC was estimated at 104% of the mean RTC hospital treatment cost, based on estimates for treating fatal and non-fatal injuries in the United States (20). After adjusting to 2022 values, the treatment costs for RTC injuries were calculated to be SAR 57,230 for minor injuries, SAR 108,329 for moderate injuries, and SAR 184,976 for severe injuries, while the cost of hospital treatment fatalities was estimated as SAR 104,425.

#### 2.2.2 Rehabilitation costs

The per patient cost of rehabilitation services for RTCs after being discharged from the KAMC hospital have been previously estimated by injury severity level (ISS 0–15, ISS 16–25, and ISS > 25) (8). These costs include laboratory costs, radiology costs, healthcare provider costs, rehabilitation visits, prosthetics/orthotics, medication costs, and bed utilization for 1 year following discharge from RTC-related hospitalization. One study estimated that 67% of patients injured in RTCs require long-term rehabilitation services (21). Based on that, we multiplied 67% of injured patients in each injury category by the per patient rehabilitation costs and aggregated the results for the total rehabilitation cost. The mean rehabilitation costs per injury in 2022 values were calculated to be SAR 15,603 for minor injuries, SAR 29,499 for moderate injuries, and SAR 31,547 for severe injuries.

#### 2.2.3 Property damage costs

The cost of property damage resulting from RTCs has been previously estimated for crashes resulting in fatalities, in serious injuries (ISS ≥ 15), minor injuries (ISS ≤ 14), and in only property damage in Saudi Arabia (22). The total cost of RTC property damage was estimated by multiplying these per crash cost estimates by the number of crashes for each severity level as reported by the General Directorate of Traffic for different crash types, as well as from a private insurance company (NAJM) for crashes resulting in property damage only (22). The per crash costs of property damage in 2022 values were calculated to be SAR 39,164 for fatal crashes, SAR 28,830 for crashes resulting in injuries with an ISS ≥ 15, SAR 26,888 for crashes resulting in injuries with an ISS < 15, and SAR 21,136 for crashes resulting only in property damage.

### 2.3 Estimation of indirect costs at baseline

Indirect costs represent economic value that is never realized due to people being injured or killed. In this analysis, indirect costs include the economic value of premature mortality and morbidity, as well as absenteeism from work (25).

There are several methodologies for placing a value on morbidity and mortality in the health economics literature. These fall into two main categories: human capital methods and willingness-to-pay methods (26). Human capital methods value mortality or injuries based on an indicator of their economic impact, for example the average wage or GDP per capita; valuation is typically only applied to deaths and injuries among the working age population, or among employed individuals. Consequently, this method results in an underestimation of the true cost that deaths and injuries can have not only to economies but to individuals, families, and their communities. Willingness-to-pay methods seek to estimate how much individuals are willing to pay for reductions in risk for their own morbidity or mortality. The valuation resulting from this method is termed the value of a statistical life (VSL), an estimation of how much an individual would be willing to pay for a 100% reduction in their own mortality risk.

To place a value on the fatalities and injuries resulting from RTCs, we relied on a methodology proposed by iRAP, which suggests a rule-of-thumb approach that draws on and combines valuation estimates derived using both the human capital and willingness-to-pay methods. It suggests that, in the absence of country-specific estimates of the value of averted RTC fatalities, each fatality should be valued at 70 times GDP per capita and RTC injuries at 25% the value of fatalities (26). Using this methodology, the value of each fatality and injury was calculated at SAR 9.0 million (i.e., 70 x SAR 129,199) and SAR 2.3 million (SAR 9.0 million x 25%), respectively.

Lastly, absenteeism costs represent wages lost while hospitalized from RTCs. The average length of hospital stays resulting from RTCs was previously estimated in Saudi Arabia to be eight days for minor injuries (ISS 0–15), 17 days for moderate injuries (ISS 16–25), and 20 days for severe injuries (ISS > 25) (8). We calculated lost wages by multiplying the number of patients in each injury category who would be employed using labor force participation rates by the average Saudi wage. Labor force participation rate, employment rate, and wage data was as per Saudi GASTAT (18). We assumed only five of every seven days of hospitalization result in missed work based on a five-day work week.

### 2.4 Interventions to improve road traffic safety in Saudi Arabia

We assessed how scaling up three interventions to improve road traffic safety in Saudi Arabia would impact the estimated economic costs of RTCs. These are: (1) full enforcement of speed limits via mobile speed cameras, (2) full enforcement of seat belt use in cars for drivers and passengers, and (3) a graduated licensing system for new drivers. The three interventions included in this analysis were selected from a list of evidence-based interventions by local experts and stakeholders, based on the existing road traffic safety context and potential for further improvements and/or enforcement.

The level of effectiveness of enforcing speed limit and seat belt laws on reducing fatalities and injuries is drawn from a systematic review conducted by WHO to inform the WHO-Choosing Interventions that are Cost-Effective (WHO-CHOICE) model (23). Effectiveness data for graduated licensing systems is drawn from a Cochrane systematic review of the evidence (27). While the effects of a graduated licensing system would benefit passengers of new drivers and other road users involved in crashes with new drivers, effectiveness evidence is only available for new drivers and so the impacts of graduated licensing systems are calculated for teenagers only.

Current enforcement levels for speed limits and seat belt laws were taken from WHO’s 2018 Global Status Report on Road Safety. Based on a questionnaire sent to key stakeholders and approved by the Saudi Ministry of Health, the report indicates that speed limits are enforced at a level of 7 out of 10 and seat belt laws are enforced at a level of 3 out of 10. Thus, we assumed that Saudi Arabia has already obtained 70% (7 out of 10) of the possible benefit of enforcing speed limits and 30% (3 out of 10) of the possible benefit of seat belt laws. We calculated the impact of scaling up enforcement of each intervention to a 10 out of 10 level, i.e., calculating the remaining 30% possible reduction from enforcing speed limits and 70% of the possible reduction from enforcing seat belt laws. While there is a graduated licensing system in Saudi Arabia, local experts have stated that there are not restrictions on driving in the graduated system, which is the source of their effectiveness. Therefore, we calculated the full potential impact of implementing a graduated licensing system for new drivers. The full impact for the three interventions and the remaining available impact estimated for Saudi Arabia is listed in Appendix C.

To calculate the combined impact of all three interventions, we use the method proposed by iRAP, which divides the additive effects of interventions by the multiplicative effects of the interventions to determine a multiple countermeasure correction factor (28). This results in a combined effectiveness less than the sum of the individual interventions to account for potential overlap between the effectiveness of interventions.

The interventions’ impact sizes are applied to the different injury severity categories, to both sexes, and to all age groups uniformly, with the exception of graduated licensing system, which applies only to teenage drivers. To calculate the amount of health and economic savings resulting from implementing these interventions, we first apply the expected reduction in mortality and injuries. For example, by fully enforcing speed limit laws, we expect 14% fewer deaths. We then calculate the economic costs associated with the lower number of deaths. Then, we subtract the economic costs in the intervention scenario to the current economic costs under baseline scenario to estimate the amount of savings that are available from implementing the intervention.

## 3 Results

### 3.1 Economic burden of road traffic crashes

The economic burden of the 24,446 injuries and 4,555 deaths resulting from Saudi Arabia’s 1.8 million RTCs in 2022 represents a total of SAR 137.6 billion (USD 36.7 billion)—the equivalent of 3.3% of Saudi Arabia’s 2022 GDP. This comes out to SAR 76,424 (USD 20,380) per crash. Table 1 shows the economic impacts by cost component.

**Table 1:** Total costs of road traffic crashes by cost component, in SAR and USD (billions).

Costs	Cost component	Total cost billions (SAR)	Cost per crash (SAR)	Total cost billions (USD)	Cost per crash (USD)
Direct costs					
	Hospital treatment costs	2.42	1,343	0.64	358
	Rehabilitation costs	0.32	178	0.09	47
	Property damage costs	38.30	21,279	10.21	5,674
Indirect costs					
	Mortality cost	41.20	22,886	10.99	6,103
	Morbidity cost	55.27	30,707	14.74	8,118
	Absenteeism costs	0.06	32	0.02	8
Total		137.56	76,424	36.68	20,380

Direct and indirect costs represent 29.8 and 70.2% of the total costs of RTCs, respectively. Morbidity accounts for the largest cost (40.2%); the second largest cost comes from mortality (29.9%), followed by property damage (27.8%), hospital treatment costs (1.8%), rehabilitation costs (0.2%), and absenteeism costs (0.04%). Males account for the vast majority (88%) of road traffic injuries and fatalities in Saudi Arabia, and consequently account for a similar large proportion of total costs (SAR 121.5 billion [88.3%]). When looking specifically at hospital and rehabilitation costs (which combined account for 2% of RTC-related total costs), minor injuries accounted for just over two-fifths (42% of treatment costs) despite accounting for 73% of total injuries, reflective of the higher treatment costs of more severe injuries (Table 2).

**Table 2:** Treatment costs and rehabilitation costs by injury type, in SAR and USD (millions).

Injury type	Hospital treatment costs		Rehabilitation costs	
	SAR (millions)	USD (millions)	SAR (millions)	USD (millions)
ISS 0–15	1,021.31	272.35	186.00	48.95
ISS 16–25	423.71	112.99	77.08	20.28
ISS 25<	497.41	132.64	56.67	14.91
Deaths	475.65	126.84	-	-
Total Costs	2,418.08	644.82	319.74	84.14

According to data from the General Directorate of Traffic, 98% of RTCs result only in property damage (no injuries or deaths). Therefore, these types of crashes account for the vast majority of property damage costs (Table 3).

**Table 3:** Property damage costs by injury type, in SAR and USD (millions).

Injury type	Cost per crash (SAR)	Number of crashes	Total crash cost SAR (millions)	Total crash cost USD (millions)
ISS 0–15	26,888	16,897	454.31	105.57
ISS 16+	28,830	6,249	180.17	41.87
Deaths	39,164	6,213	243.33	56.55
Property damage only	21,136	1,770,641	37,423.80	9,848.37
Total Costs			38,301.62	10,079.37

### 3.2 Impact of scaling up road traffic safety interventions

The total number of lives saved in 1 year by scaling up the three interventions is estimated to be 610 and the total number of injuries averted is estimated to be 3,823. Stricter enforcement of seatbelt laws results in the largest number of lives saved and injuries averted (Table 4), reflective of the lower current level of enforcement. Because graduated licensing systems are applicable only to new drivers, injuries and deaths averted only occur in the youngest age group (<18 years). Across interventions, the age group with the highest number of lives saved and injuries averted is the 19–30-year age group, as they are the group most affected by RTCs.

**Table 4:** Number of lives saved and injuries averted from scaling road traffic safety interventions to full enforcement, by age group.

Age groups	Speed limit enforcement	Seat belt law enforcement	Graduated licensing system	Combined inter-vention package
Lives saved				
Below 18 years	27	50	92	158
19–30 years	58	106	0	160
31–40 years	49	91	0	136
41–59 years	30	56	0	84
Over 51 years	26	48	0	72
Total	191	351	92	610
Injuries averted				
Below 18 years	61	426	474	833
19–30 years	132	923	0	1040
31–40 years	116	810	0	913
41–59 years	78	545	0	614
Over 51 years	54	376	0	424
Total	440	3,080	474	3,823

Consequently, the intervention with the largest savings is full enforcement of the seatbelt law (SAR 10.56 billion), followed by full speed limit enforcement (SAR 2.81 billion), and graduated licensing system (SAR 1.96 billion) (Table 5). Scaling up these three road safety interventions could avert 10.65% of the total economic burden resulting from RTCs. The SAR 14.65 billion in savings when combining the interventions would be equivalent to 0.35% of Saudi Arabia’s GDP in 2022.

**Table 5:** Total economic savings from scaling up road traffic safety interventions to full enforcement, by cost component in SAR (millions).

Type of cost averted		Savings by intervention			
		Speed limit enforcement	Seat belt law enforcement	Graduated licensing system	Total savings
Direct	Hospital treatment costs	54.94	281.37	26.36	303.79
	Rehabilitation costs	5.76	40.29	4.34	50.01
	Property damage costs	21.65	98.69	12.30	129.06
Indirect	Mortality cost	1,730.19	3,172.02	828.91	5,519,61
	Morbidity cost	994.89	6,964.26	1,071.36	8,636.85
	Absenteeism costs	1.03	7.18	-	8.09
Total Costs Averted		2,808.46	10,563.80	1,956.42	14,647.41

### 3.3 Sensitivity analysis

We conducted a sensitivity analysis using a higher number of RTC injuries based on the rule-of-thumb approach suggested by iRAP of 10 injuries per one RTC fatality and/or a higher number of RTC fatalities based on the estimates provided by WHO.

Thus, if the iRAP recommended 10:1 ratio for injuries to fatalities is applied to the Saudi Arabia’s reported number of fatalities (i.e., 4,555), the economic cost of RTCs is calculated to be SAR 187.23 billion (USD 49.93 billion). This would represent a 36.1% increase in the economic cost based on the currently reported number of deaths and injuries, and would correspond to 4.5% of Saudi Arabia’s GDP. While the total number of fatalities averted would remain the same (i.e., 610), the total number of injuries averted by the combined interventions would increase to 7,149 (from 3,823). This would increase the total savings from implementing the three interventions to SAR 22.47 billion, the equivalent of 0.54% of Saudi Arabia’s 2022 GDP.

When using WHO’s mortality estimate for RTC fatalities in Saudi Arabia (i.e., 6,735) and applying the recommended 10:1 ratio for injuries to fatalities, the economic cost of RTCs is calculated to be SAR 258.48 billion (USD 68.93 billion). This would represent a 87.9% increase in the economic cost based on the currently reported number of deaths and injuries, and would correspond to 6.2% of Saudi Arabia’s GDP. The total number of fatalities and injuries averted by the combined interventions would increase to 902 (from 610) and 10,570 (from 3,823), respectively. This would increase the total savings from implementing the three interventions to SAR 33.07 billion, the equivalent of 0.80% of Saudi Arabia’s 2022 GDP.

## 4 Discussion

This study estimates the economic cost from RTCs in Saudi Arabia and analyzes total economic savings from scaling up a set of three road traffic safety interventions, namely speed limits, seat belts and graduate licensing. Despite improvements in road traffic safety since 2016, RTCs continue to represent an important economic burden, equivalent to 3.3% of the country’s 2022 GDP. If WHO mortality data and the iRAP 10:1 injury to fatality ratio were to be used, then this figure could be as high as 4.6%. These figures are higher than existing estimates of the economic costs of RTCs, both for Saudi Arabia (e.g., a previous analysis of RTCs in Saudi Arabia put the economic cost at 2.9% of GDP) (22) and other countries (e.g., Iran, Nepal, U.S.A.) (29–31). Incidentally, WHO reports that most countries lose the equivalent of 3% of their GDP to RTCs (3). This difference in the economic impact (as it relates to GDP) is most likely a reflection of the high number of RTC-related fatalities and injuries that still occur in Saudi Arabia and/or driven by the use of lower valuations of the value of averted mortality and morbidity.

Estimates of the economic costs that are limited to only direct cost components underestimate the full economic effect of road traffic crashes. Thus, in our analysis indirect costs accounted for a large proportion of total cost, 70.2%, compared to 29.8% for direct costs. Even so, direct medical costs can still impose immediate and sometimes unsustainable burden on health systems.

Our analysis demonstrates that the economic cost to Saudi Arabia’s society could be reduced with scaling up and fully enforcing road traffic safety interventions already in place. The results underscore the need to support full enforcement of existing laws to reduce the economic impacts of RTCs. For example, seatbelt laws have been in place in Saudi Arabia for over 20 years, yet prevalence of seatbelt wearing and use remains poor [e.g., in 2013 it was reported to be as low as 5% (32, 33)]. In addition to complimentary “Safe System” interventions (34) that were rolled-out in the past few years (e.g., establishment of an automated traffic enforcement system for serious violations, expansion of regulations related to vehicle safety and unsafe parking, and strengthening the post-crash response) (9, 11), Saudi Arabia should consider other approaches and interventions to reduce the burden and economic impact of RTCs—these could include heightened enforcement through alternative means (e.g., artificial intelligence-based surveillance), prioritization of high-risk groups (i.e., male drivers <30 years of age), and implementation of more expansive and intensified public awareness campaigns. To do so, Saudi Arabia’s Vision 2030, which prioritizes improving road safety, will need to be accompanied by more resources to implement the full range of the WHO-recommended road safety measures (14) to build on progress made to date.

That reducing RTC-related deaths and injuries is possible with the right political commitment, resources and implementation and enforcement of interventions, is the fact that between 2010 and 2021 ten countries (Belarus, Brunei, Denmark, Japan, Lithuania, Norway, Russian Federation, Trinidad and Tobago, United Arab Emirates and Venezuela) succeeded in reducing RTC-related death by 50% (2).

Our study has several limitations. First, our source for the number of injuries and fatalities in Saudi Arabia, the Ministry of Health, reports deaths and injuries that are significantly lower than global databases, such as the WHO Global Health Observatory and the GBD study. While it is known that there are systemic gaps in Saudi injury surveillance, (35) the study team felt it was important to use locally-available data; due to the importance given to RTCs in Saudi Arabia’s Vision 2030, we believe that injury surveillance systems and approaches will improve. Yet, to mitigate for the likely underestimate using the currently available data, we also calculated the costs using WHO Global Health Observatory figures, showing how both the economic costs as well as the economic savings due to the interventions would change if these figures were used. Second, the estimates of RTC injuries from the Ministry of Health do not include those injuries that do not require hospitalization, those that are hospitalized for less than 24 h, or for those services not covered by the public health system. This likely results in a substantial underestimation of the total cost due to RTCs. To address this limitation, we conducted a sensitivity analysis with a higher number of injuries (i.e., using the iRAP recommended 10:1 ratio of injuries to fatalities). Third, treatment cost estimates used for minor, moderate, and severe injuries were based on a single study with a comparatively small sample size (20). Treatment costs for patients treated and discharged on-the-spot were not included in that study. No data for these patients with very minor injuries was available at the time of the analysis, and creating a separate costing mechanism for these patients was beyond the scope of the study. Fourth, we rely on rule-of-thumb estimations for the valuation of mortality and morbidity in the absence of country-specific estimates. Country-specific estimates of the willingness to pay for reductions in mortality and morbidity would improve the accuracy of results. Last, while cost-of-illness studies have played a significant role in public health by supporting advocacy for various policies, their usefulness in decision making for prioritization and resource allocation needs to be complemented with a consideration of both costs and benefits.

## 5 Conclusion

Our findings demonstrate the continued economic burden associated with RTCs in Saudi Arabia. These findings could be used by policymakers to advocate for more resources to reduce the number of RTC-related injuries and deaths, as well as to assess options for enhancing the enforcement of existing road traffic safety laws and to prioritize additional interventions and approaches. Given the health and economic burden of RTCs in Saudi Arabia, further studies should be conducted, in both public and private health facilities, to more accurately estimate the true number of RTC-related injuries and deaths, as well as associated individual, facility and societal economic costs.

## Data Availability

The original contributions presented in the study are included in the article/Supplementary material, further inquiries can be directed to the corresponding author.
