# Supplementary material for: Assessing the economic cost of road traffic crashes in Saudi Arabia: potential savings from scaling up interventions
Source: Front Public Health. 2025 Sep 23;13:1637609. doi: 10.3389/fpubh.2025.1637609 (PMC12502079; doi:10.3389/fpubh.2025.1637609)
Supplement: Supplementary file 1 [file Supplementary_file_1.pdf]

Assessing the economic cost of road traffic crashes in Saudi Arabia: potential savings from scaling up interventions

Appendix

Garrison Spencer[1], Mohammed Alluhidan[2], Richard Reithinger[1], Reem F. Alsukait [3], Radoslaw Czapski [3], Adwa Alamri[2], Volkan Cetinkaya [3], Fatimah Alshehri[4], Said Dahdah[3], Mariam M. Hamza [3], Christopher H. Herbst [3], and Suliman Alghnam[5]

1: RTI International, Washington, D.C., USA; 2: Department of Health Economics Saudi Health Council, Riyadh, Saudi Arabia; 3: World Bank Group, Washington, D.C., U.S.A.; 4: Department of Injury Prevention, Saudi Public Health Authority, Riyadh, Saudi Arabia; 5: Public Health Intelligence, Saudi Public Health Authority, Riyadh, Saudi Arabia.

Annex A: Summary of data sources

Parameter	Description	Source
Country demographic and economic parameters		
Saudi Arabia population	2022 Saudi Arabian population	GASTAT [18]
GDP	2022 Saudi Arabian GDP	GASTAT [18]
Consumer Price Index	Annual Saudi Arabian consumer price index	GASTAT [18]
Mortality and morbidity parameters		
Mortality due to RTC	Number of deaths from RTCs	Ministry of Health [13]
Morbidity due to RTC	Number of injuries from RTCs	Ministry of Health [13]
Cost parameters		
Hospital treatment costs	Average treatment costs of injuries based on injury type (i.e., light, moderate, severe)	Alghnam et al. [19]
Cost of dying in hospital	Average treatment costs of RTC case succumbing to injuries	Peterson, et al. [20]
Rehabilitation costs	Average costs for rehabilitation based on injury severity	Alghnam et al. [8]
Duration of injury	Average length of stay in hospital	Alghnam et al. [21]
Labor force participation rate	Labor force participation rate used to adjust income loss	GASTAT [18]
Daily wages	Estimated from mean monthly wage for Saudi Arabia	GASTAT [18]
Property damage costs	Estimated property damage based on injury type	Alarifi et al. [22]
Intervention parameters		
Effectiveness of RTC intervention	Effectiveness of RTC interventions	Chisholm & Naci [23]
Others		
Value of Statistical Life	Used in estimating the economic value of deaths	Green [24]
Value of Morbidity	Used in estimating the economic value of morbidity	Green [24]

Annex B: Number of RTC injuries, by injury severity score and sex

	Total	Male	Female
Minor injuries (ISS 0–15)	17,846	15,758	2,087
Moderate injuries (ISS 16–25)	3,911	3,454	457
Severe injuries (ISS >25)	2,689	2,375	315
Total injuries	24,446	21,587	2,859

Annex C: Intervention effectiveness for road traffic injuries and mortality

	Speed limit enforcement	Seat belt enforcement	Graduated licensing system
Total intervention effect size on non-fatal outcomes	-6.0%	-18.0%	-14.0%
Intervention effect size expected in Saudi Arabia on non-fatal outcomes, accounting for current level of implementation	-1.8%	-12.6%	-14.0%
Total intervention effect size on mortality	-14.0%	-11.0%	-14.0%
Intervention effect size expected in Saudi Arabia on mortality, accounting for current level of implementation	-4.2%	-7.7%	-14.0%
